# The origin of B chromosomes in yellow-necked mice (*Apodemus flavicollis*)—Break rules but keep playing the game

**DOI:** 10.1371/journal.pone.0172704

**Published:** 2017-03-22

**Authors:** M. Rajičić, S. A. Romanenko, T. V. Karamysheva, J. Blagojević, T. Adnađević, I. Budinski, A. S. Bogdanov, V. A. Trifonov, N. B. Rubtsov, M. Vujošević

**Affiliations:** 1 Department for Genetic Research, Institute for Biological Research “Siniša Stanković“, University of Belgrade, Belgrade Serbia; 2 Institute of Molecular and Cellular Biology, SB RAS, Novosibirsk Russia; 3 Institute of Cytology and Genetics, SB RAS, Novosibirsk Russia; 4 Koltzov Institute of Developmental Biology, RAS, Moscow Russia; Leibniz-Institute of Plant Genetics and Crop Plant Research (IPK), GERMANY

## Abstract

B chromosomes (Bs) are known for more than hundred years but their origin, structure and pattern of evolution are not well understood. In the past few years new methodological approaches, involving isolation of Bs followed by whole DNA amplification, DNA probe generation, and fluorescent *in situ* hybridization (FISH) or the B chromosome DNA sequencing, has allowed detailed analysis of their origin and molecular structure in different species. In this study we explored the origin of Bs in the yellow-necked wood mouse, *Apodemus flavicollis*, using generation of microdissected DNA probes followed by FISH on metaphase chromosomes. Bs of *A*. *flavicollis* were successfully isolated and DNA was used as the template for B-specific probes for the first time. We revealed homology of DNA derived from the analyzed B chromosomes to the pericentromeric region (PR) of sex chromosomes and subtelomeric region of two pairs of small autosomes, but lower homology to the rest of the Y chromosome. Moreover, all analysed Bs had the same structure regardless of their number per individual or the great geographic distance between examined populations from the Balkan Peninsula (Serbia) and Eastern Europe (south region of Russia and central Belarus). Therefore, it was suggested that B chromosomes in *A*. *flavicollis* have a unique common origin from the PR of sex chromosomes, and/or similar evolutionary pattern.

## Introduction

As the oldest known and most frequently occurring polymorphism, that of B chromosomes (Bs) remains intriguing from many aspects after more than a century of research. B chromosomes are defined as dispensable supernumerary chromosomes, that do not recombine with members of the basic A chromosome complement and do not follow the rules of the Mendelian segregation law [1]. In the last few decades the opinion that Bs are selfish, junk or parasitic elements dominated [2, 3]. Similar terms were used to describe noncoding sequences, which make up most of the genome. They were considered to be non-functional DNA, but nowadays different experimental approaches have strongly disproved this assumption by showing that noncoding RNAs play an important role in regulation of genome function [4–9]. Analogous changes of view concerning Bs are happening currently, as knowledge of their structure increases [10].

Bs are present in all main groups of fungi, plants and animals [11] except birds [12]. However, we could not exclude that in birds the Bs were hidden among the numerous microchromosomes. About 15% of all explored eukaryotes have Bs, but only 1.5% of mammalian species possess them [12, 13]. Within mammals Bs are most frequent in rodents, which account for 70% of mammalian species harbouring them [14]. Variation of their occurrence goes in many directions. Namely, they may be present in all populations of some species or in just a few of others. Their frequency and number also varies between or within populations of the same species. Differences also persist in single specimens where Bs could be absent from some organs. Furthermore, variability may produce tissue mosaicism, which is often a feature of mammalian species. In general Bs are equally distributed between the sexes, but departure from the one-to-one ratio is also documented. Morphology of Bs is also very diverse when both inter- and intra-species variations are considered [15]. Generally, it was concluded that Bs do not recombine with chromosomes from the A set [2]. However, molecular evidence of meiotic recombination has been found for Bs in the silver fox, *Vulpes vulpes* [16].

Bs were observed to occur more frequently in species with acrocentric chromosomes in their karyotype [17, 18]. The general consideration is that Bs originated either from autosomes or sex chromosomes (A set) of the same species, or from a sister species as a consequence of interspecies hybridization. The hypotheses on the B chromosome origin were reviewed by Camacho et al. [2]. Many studies in last few years indicate that Bs are actually composed of segments from different chromosomes of the A set [10, 19, 20]. Bs in species from the tribe Oryzomyini, originated from the pericentromeric heterochromatin region of the sex chromosomes [21]. In the Korean field mouse, *Apodemus peninsulae*, Bs originated from both autosomal and sex chromosome heterochromatin [22]. Furthermore, variability in DNA composition of Bs in different geographical regions indicate difference in origin of Bs in distinct populations of this species [22, 23]. In the fruit fly *Drosophila albomicans*, Bs may have arisen as a fusion product of different chromosomes [24]. The B in rye, *Secale cereale*, is a mosaic of A chromosome and organellar sequences [19]. Some Bs variants in maize, *Zea mays*, may have arisen from unequal crossing over of B chromosomes [25]. In the cichlid fish, *Astatotilapia latifasciata*, Bs are composed of sequences arising from gene duplication of almost all autosomes and modified by mobile elements or retrotransposon insertions [10]. In grasshopper, *Podisma sapporensis* cluster of segmental duplication in some cases could be refered to proto-B chromosome or process of B formation during its evolution [26].

Early studies of the genetic structure of Bs in different species involved fluorescent *in situ* hybridization (FISH) of available probes for repetitive elements. In this way, different repetitive elements were localized on Bs in many species [27–30]. Also, some autosomal genes were found on Bs using BAC clone probes [31]. In order to explore the origin and complete molecular content of Bs in detail, only isolation of those elements either by flow cytometry or microdissection, followed by FISH and sequencing should be used. Thus, use of B-specific probes derived by microdissection in the FISH approach has confirmed the mostly repetitive content and revealed the origin of Bs in some cases [32, 21].

Next generation sequencing has complemented our knowledge about the molecular structure of Bs in different species. Thus, Bs are now regarded as a collection of accumulated repetitive elements interrupted by sequences that are homologous to gene fragments or even complete genes [13, 19]. In some cases Bs have a higher density of repetitive elements than the autosomes [29, 33], especially 18S rRNA genes. Interesting findings are that some genes present on Bs in different species belong to the group regulating the cell cycle [10, 13]. Furthermore, transcriptional activity has been shown for some of them [13, 34, 35]. The appearance and transcriptional activity of cell cycle regulation genes on Bs in different species could be the key of their maintenance.

Despite progress in collecting evidence about their origin, the gene content and pattern of evolution of Bs are still open questions for the majority of species. One of them is the yellow-necked wood mouse, *Apodemus flavicollis*. In this species, Bs were found in almost all studied populations [36–39]. The standard karyotype (2n = 48) can harbour additionally up to eight B chromosomes [40]. The additional elements were described as acrocentric, the same size as the smallest autosome pairs or rarely, even smaller micro-Bs [41, 42]. In this species Bs do not recombine with chromosomes from the A set [43, 44]. There is no direct evidence concerning the gene content of Bs in these mice, but several studies have shown differences in either gene expression or gene copy number in individuals possessing B chromosomes. Differential expression of three cDNA fragments was detected in mice with Bs compared to those without them [45, 46]. Moreover, individuals with Bs were found to have lower expression levels of the Tgf-β gene [47]. Higher copy numbers of the 18S rRNA gene were detected in one sample with two Bs, while other samples with one, two or three Bs did not exhibit differences in copy number in comparison to control samples without Bs [48]. In spite of our partial knowledge about the structure of Bs in this species, population studies have revealed the presence of subtle effects of Bs at different levels [49–53].

In the present study we provide the first evidence of the origin and structure of Bs in *A*. *flavicollis* by means of microdissection of B chromosomes and FISH mapping.

## Material and methods

### Specimens

All animals used in this study were collected using Longworth traps and treated according to the legal and ethical guidelines as indicated in Directive 2010/63/EU of the European Parliament and the Council of 22nd September 2010 on the protection of animals used for scientific purposes. Anesthetic overdose with prior sedation was used for mouse euthanasia. All efforts were made to minimize animals suffering. All animal procedures were approved by the Ethical Committee for the Use of Laboratory Animals of the Institute for Biological Research “Siniša Stanković”, University of Belgrade. A total of 9 specimens of *Apodemus flavicollis* from different populations and with different number of B chromosomes were used in this study. Samples were collected from five localities: Minsk region, Republic of Belarus (N 53° 57’, E 27° 48’), Rostov-on-Don region, Russia (N 47° 38’, E 41° 56’), Milošev Do, Serbia (N 43° 18’, E 19° 47’), Petnica, Serbia (N 44° 14’, E 19° 55’), Orašac, Serbia (N 44° 19’, E 20° 35’). All localities are beyond protected areas or private lands, except Nature Park Donskoy in Minsk region, where specimen 24985 was captured, which formally permits conduction of scientific investigations and ecological monitoring on the territory (according to Resolution № 389 of Administration of Rostov-on-Don region).

The yellow-necked mouse *Apodemus flavicollis* is not endangered or protected species in Serbia, Belarus Republic and Russia. The species is not included in Red lists of Serbia, Belarus and Russia. Three samples were used for microdissection of B chromosomes (Table 1). Labeled probes were hybridized onto chromosome preparations made from eight specimens (Table 2). Chromosome preparations were made either from old chromosome suspensions of bone marrow cells and gonad tissue or from newly established primary fibroblast cell cultures.

**Table 1:** List of samples used for microdissection of B-like chromosomes.

Sample	Labelled B-specific probe	Karyotype	Preparation made from	Locality
3727	3727WCPB	48,XY,+1B	testicular tissue	Milošev Do, Serbia
3980	3980WCPB	48,XX,+1B	fibroblast cell culture	Orašac, Serbia
24985	24985aWCPB24985bWCPB	48,XX,+3B	bone marrow	Rostov-on-Don region, Russia

**Table 2:** List of samples used for hybridization.

Sample	Karyotype	Preparation made from	Locality
3980	48,XX,+1B	Primary cultured fibroblasts	Orašac, Serbia
3977	48,XX,+1B	Primary cultured fibroblasts	Petnica, Serbia
3978	48,XY	Primary cultured fibroblasts	Orašac, Serbia
3979	48,XY	Primary cultured fibroblasts	Orašac, Serbia
3656	48,XX,+2B	Cells of bone marrow	Milošev Do, Serbia
3854	48,XX,+3B	Cells of bone marrow	Misača, Serbia
24985	48,XX,+3B	Cells of bone marrow	Rostov-on-Don region, Russia
24943	48,XY,+1B	Cells of bone marrow	Minsk region, Republic of Belarus

### Cell cultures

Primary fibroblast cultures were established from four animals from central Serbia. The choice was based on the presence of Bs marker, using the ISSR-PCR method [54]. Two females with the B-specific marker and two males without it were selected. Tail portions from each animal were wiped with 70% ethanol, cut off and immersed in physiological saline solution containing antibiotics (penicillin 500,000 U/l and kanamycin 500 mg/l) and an antimycotic (amphotericin B 12.5 mg/l). Skin was removed from the pieces of tail and rinsed three times with fresh amounts of the same physiological solution, all under sterile conditions.

Fibroblast cell cultures were obtained using the protocol of [55] as modified by [56]. In our study cells were cultivated at 37°C and 5% CO_2_. Depending on the rate of cell growth, the nutritive medium containing the antibiotics/antimycotic mixture was changed every few days. Cells were passaged when they covered the flask surface completely. Affixed cells were loosened off with 0.25% trypsin, 0.2% EDTA or scraped free. After several passages the amount of cells was sufficient for chromosome preparation.

### Metaphase chromosome preparation

Chromosome preparations from bone marrow and meiotic cells were obtained by the standard technique [57, 58]. The presence and number of Bs were determined from twenty analysed metaphase figures. All animals with more than 48 chromosomes (standard complement) were considered to have Bs. Chromosome preparations were also performed from established fibroblast cell cultures as follows. After adding colcemid (0.04 μg/ml) cells were kept in a CO_2_ controlled incubator at 37°C overnight followed by incubation with EtBr (1.5 μg/ml) for 3 hours before harvesting. Hypotonic solution (33.5 mM KCl, 7.75 mM sodium citrate) was then added and the cells incubated for 55 minutes at 37°C. Chromosomes were prefixed and fixed according to standard protocol with fresh prepared ice cold fixative (methanol and glacial acetic acid in ratio 3:1). Slides for preparation were well washed and preserved at 4°C in distilled water. One drop of chromosome suspension was spread on cold, clean and wet slide and dried. Chromosomes were stained with Giemsa and analysed under a microscope. The presence and number of Bs was determined as previously described.

### GTG banding of metaphase chromosome

GTG-banding of metaphase chromosomes were performed according to the standard protocol [59].

### Generation of microdissected DNA libraries and painting probes derived from the B chromosomes

In order to obtain B-specific probes, four B-like chromosomes from the chromosome preparations of three B-carrying individuals were microdissected and each transferred to individual tubes as described by Yang et al. [60]. The microdissected material was amplified using a whole genome amplification kit (WGA SIGMA-ALDRICH, USA) according to the manufacturer’s protocol, which allows amplification of fragments of 300bp to 3000 bp.

The amplified PCR product was labelled in Genome Amplification kit (WGA SIGMA-ALDRICH, USA) with an additional fluorescent labelled nucleotide, Tetramethyl-Rhodamine-5-dUTP or biotin 16-dUTP. The names of DNA probes derived from the Bs contained number of specimen and followed with WCPB abbreviation (Whole Chromosome Paint for the B) (Table 2). Painting probes derived from the Bs of specimen 24985 were named 24985aWCPB and 24985bWCPB. B-specific probes were labelled as follows: the 3727WCPB and 3980WCPB were labelled with biotin-16-dUTP, while 24985aWCPB and 24985bWCPB were labelled with tetramethyl-Rhodamine-5-dUTP. Specificity of obtained painting probes were estimated with FISH on the metaphase chromosomes used for DNA probe generation.

### *Mus musculus* painting probe for the whole X chromosome and DNA probe derived from bacterial artificial chromosome containing mouse X-specific DNA fragment

Besides B-specific probes, standard fluorescent probes were evaluated as controls using FISH. We tested hybridization of the BAC clone X-specific fluorescent probe and *Mus musculus* whole chromosome X labeled probe (XCyting Muse Chromosome Painting Probes, MetaSystems, Altlussheim).

### Fluorescent *in situ* hybridization (FISH)

For all tested probes FISH procedures were done according to the protocol of Trifonov et al. [61]. The chromosomes were counterstained with 4’,6-diamidino-2-phenylindole dihydrochloride (DAPI) and analysed under a fluorescence microscopes Olympus BX53 and Axioskop 2 plus (Zeiss). FISH was also carried out on previously GTG-banded chromosomes. Images of GTG-banded chromosomes were captured, coverslip was removed with xylol. Slides were dried and rinsed in fixative. Then FISH was performed according standard procedure. FISH with *Mus musculus* WCPX and with the mouse X-specific DNA probe were carried out as described in manufacturer’s protocols.

## Results

**Fig 1:**
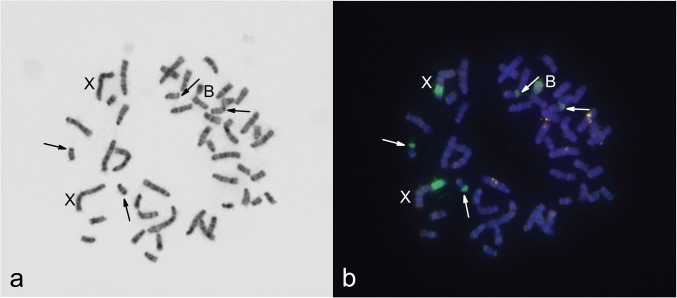
Metaphase plate from fibroblast cell culture from a female with one B (3980). (**a**) G-band staining; (**b**) hybridisation of 3727WCPB (green signal). B chromosome and X chromosomes are marked, arrows indicate heterochromatic blocks on two pairs of small autosomes. DAPI is blue.

**Fig 2:**
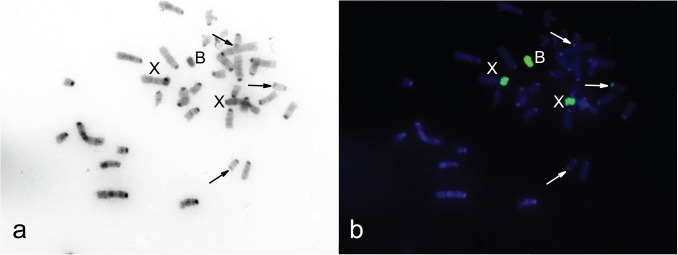
Incomplete metaphase plate of sample 3980. (**a**) Inverted DAPI staining; (**b**) hybridisation of 3980WCPB (green signal). B chromosome and X chromosomes are marked, arrows indicate heterochromatic blocks on three small autosomes.

For karyotyping of the specimens involved in this study, cultures of primary fibroblasts and cells of bone marrow were used. Up to three Bs were found in cells of seven specimens, while two specimens contained no Bs. All Bs were acrocentrics with the same size as small As. Identification of Bs was allowed by application of GTG- and DAPI-banding of metaphase chromosomes (Fig 1A). Both techniques stained Bs almost homogenously (Fig 2A). DAPI stained the Bs brighter than most C-negative regions but darker than C-positive regions of As, (pericentromeric regions of autosomes, large proximal C-positive regions of the X, and subtelomeric region in two pairs of small autosomes).

All obtained DNA probes painted completely the original Bs and some additional regions of A chromosomes (Fig 1A and 1B).

During preparation of chromosome metaphases for FISH (sample 3980 with one B), application of the G banding technique revealed one small heterochromatic chromosome (Fig 1A). DAPI staining performed on a different metaphase from the same specimen (Fig 2A), in accordance with C banding, revealed the presence of constitutive heterochromatin as a bright region near the centromere of all chromosomes except for a small one, which was completely luminous. Hybridization of B-specific probes (3980WCPB and 3727WCPB) confirmed that the small heterochromatic elements were Bs (Fig 1B, Fig 2B). Besides B chromosomes, hybridization signals were observed at the PR of two large chromosomes and subtelomeric regions of two pairs of small chromosomes from the A set (Fig 1B, Fig 2B).

**Fig 3:**
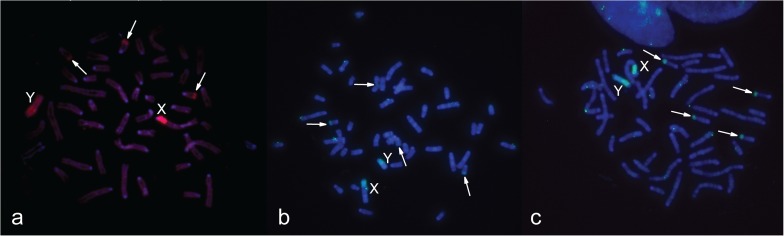
Hybridisation of different B-specific probes onto male metaphases without Bs from one population, (**a**) 24985aWCPB (red signal); (**b**) 3980WCPB (green signal); (**c**) 3727WCPB (green signal). Y and X chromosomes are marked, arrows indicate heterochromatic blocks on four small autosomes.

The strong signal of 24985aWCPB, 3980WCPB and 3727WCPB hybridization to different male metaphases without B chromosomes confirmed high homology of the all tested WCPBs to the PR of sex chromosomes, and lower homology with the rest of the Y chromosome. In all studied cases, a hybridization signal was also present at the subtelomeric region of four small chromosomes from the A set (Fig 3A, 3B and 3C).

**Fig 4:**
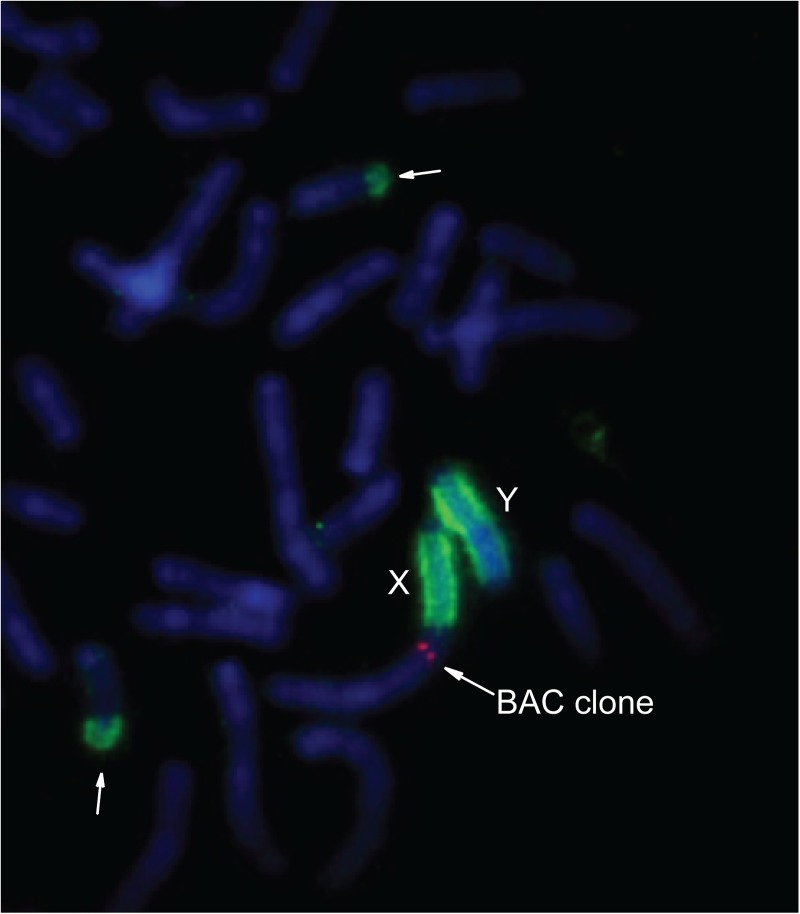
Incomplete metaphase of sample 3978 hybridized with 24985bWCPB (green signal) and X-specific BAC clone probe (red signal). Y chromosome, X chromosome and BAC clone are marked, arrows indicate heterochromatic blocks on two small autosomes. DAPI is blue.

**Fig 5:**
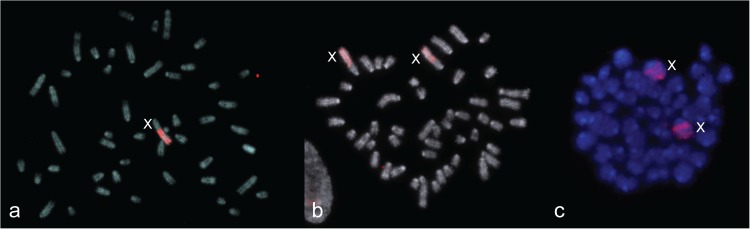
Hybridization of *Mus musculus* whole chromosome X-specific probe (red signal) onto *A*. *flavicollis* metaphases with and without B chromosomes. (**a**) metaphase of a zero B male (3978); (**b**) metaphase of a one B female (3980); (**c**) metaphase of a three B female (3854); X chromosomes are marked. DAPI is grey (**a**, **b**) and blue (**c**).

Simultaneous hybridization of the X-specific probe (BAC clone) and 24985bWCPB to preparations of male metaphase without Bs (3978), confirmed the similarity of Bs to the PR of sex chromosomes (Fig 4). Hybridization of the *Mus musculus* whole chromosome X specific probe to the X chromosome in the analysed samples, showed a strong signal along the whole length excluding the PR (Fig 5A and 5B). We did not detect a hybridization signal of the *Mus musculus* whole chromosome X-specific probe on Bs in preparations from samples of female individuals either with one B (Fig 5B) or three Bs (Fig 5C).

**Fig 6:**
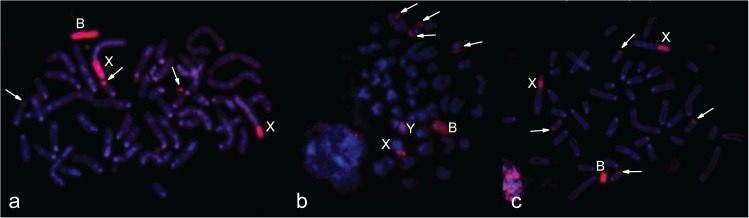
Hybridization of WCPBs onto metaphases with a one B chromosome from distinct populations of *A*. *flavicollis*. (**a**) 24985aWCPB (Rostov-on-Don region, Russia) (red signal) hybridized onto 3980 (Orašac, Serbia); (**b**) 24985bWCPB (Rostov-on-Don region, Russia) (red signal) hybridized onto 24943 (Minsk region, Republic of Belarus); (**c**) 24985bWCPB (Rostov-on-Don region, Russia) (red signal) hybridized onto 3977 (Petnica, Serbia); B chromosomes and X chromosomes are marked, arrows indicate heterochromatic blocks on four small autosomes. DAPI is blue.

In order to check the origin of Bs, different combinations of probes and preparations were used in FISH from distinct populations of *A*. *flavicollis*. Two WCPBs, 24985aWCPB and 24985bWCPB (from the Rostov-on-Don region, Russia), were hybridized to preparations of samples with one B, from central Serbia (Fig 6A and 6C) and the Minsk region, Belarus (Fig 6B) in order to detect possible diversity related to population origin. Hybridized probes showed the same affinity to B chromosomes, the PR of sex chromosomes and the subtelomeric region of four small autosomes, despite geographically distant sample origins. Due to disparity in preparation quality and chromosome size, small signals on the subtelomeric region were sometimes hardly visible in chromosome preparations from bone marrow.

**Fig 7:**
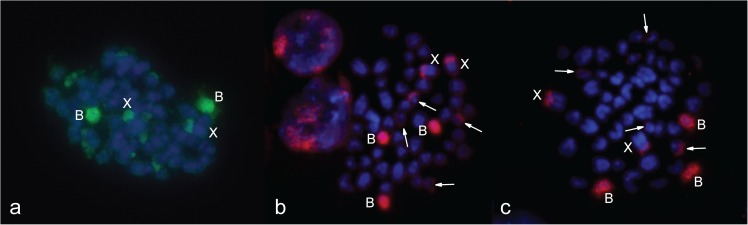
Hybridization of WCPBs onto metaphases with more than one B chromosome. (**a**) 3980WCPB (green signal) hybridized onto 3656 (2B); (**b**) 24985aWCPB (red signal) hybridized onto 3854 (3B); (**c**) 24985bWCPB (red signal) hybridized onto 24985 (3B); B chromosomes and X chromosomes are marked, arrows indicate heterochromatic blocks on four small autosomes. DAPI is blue.

Results of probe hybridization to sample preparations with more than one B chromosome from geographically distinct populations confirmed the same homology of the WCPB to all Bs present in metaphase. Thus, the preparation from the two B female samples from south-western Serbia hybridized with the 3980WCPB and, despite the poor chromosome spread, two B chromosomes and two X chromosome signals were visible (Fig 7A). Hybridization of the 24985aWCPB to the three B female sample from central Serbia (Fig 7B), as well as that for the 24985bWCPB to the three B female sample from a Russian population (Fig 7C), revealed that the same WCPB is homologous to all three Bs, the PR of sex chromosomes and the subtelomeric region of two pairs of small autosomes.

## Discussion

During evolution, the rodent karyotype has passed through many chromosomal rearrangements [62], so it is not surprising that among mammalian species with Bs 70% are rodents [14]. About 30% of species in the genus *Apodemus* contain Bs [36, 38, 63].

To clarify the question on the B chromosome molecular organization in *A*. *flavicollis* four microdissected DNA libraries were generated and used in FISH experiments. Our results unequivocally indicate homology of the B-specific sequence to the PR of sex chromosomes, lower homology with the rest of the Y chromosome, but strong homology to the subtelomeric region of two pairs of small autosomes.

The origin of Bs from sex chromosomes has been demonstrated in a number of species [21, 64, 65]. Considering G- and C-staining, the region of sex chromosomes in *A*. *flavicollis* to which our B-specific probe hybridized was described differently. The PR of the X chromosome in *A*. *flavicollis*, that showed homology to the B-specific probe was marked as a heterochromatic region based on the C-band [39], but was stained half light/half dark according to the G-band (Fig 1A). In contrast, the PR of chomosome Y that is homologous to the B-specific probe is C-negative [39]. This region was characterized in two ways considering the presence of repetitive elements and meiotic behaviour.

Rubtsov et al. [66] found that the region below the centromere on the X chromosome in *A*. *flavicollis*, is homologous to microdissected pericentromeric repetative sequences that are present on all chromosomes. Since our B-specific probe did not hybridize onto the PR of autosomes, we suppose that the DNA sequence of the B chromosome is different from the autosomal PR. Equal homology of sex chromosome PRs to both the B sequence and an autosomal PR probably represent a combination of these two types of sequences in this region.

In adittion, absence of signal on PR of X chromosomes as well as on Bs in *A*. *flavicollis* after applying of *Mus musculus* whole chromosome X-specific probe, could indicate that Bs originate from a region of the sex chromosome in *A*. *flavicollis* that does not exist on the X chromosome in *Mus musculus*.

Another view regarding the PR of chromosomes X and Y was obtained by studies of male meiosis. The ends of these chromosomes were confirmed as regions of meiotic recombination (PAR) in *A*. *flavicollis* [67]. This region of chromosome X and Y that pair in pachytene of male meiosis was shown to be next to the centromere in *A*. *sylvaticus* [68] and *A*. *flavicollis* [39].

The PAR of sex chromosomes is a small euchromatic region homologous between X and Y. Recombination in PAR is about ten more often in male than in female meiosis and at least twenty times higher than in autosomes [69–71]. Since double stranded breaks are more frequent in PAR than in the rest of the genome, together with presence of the centromere, makes this region of the sex chromosomes a good candidate for neo-B.

According to our results, the PR of sex chromosomes is the most probable ancestor of all Bs in *A*. *flavicollis*. Regardless of heterochromatinisation, we can confirm the mostly repetitive nature of the DNA sequences that form this part of the sex chromosomes [66]. We do not have direct evidence that this region contains the PAR-related genes, but the meiotic behaviour of X and Y suggests that PAR could interrupt this region or be its boundary and a place of breakage.

Besides the bright signal of B-specific probe hybridization on the PR, we found a lower hybridization signal on the rest of the Y chromosome, as well as a strong one on the subtelomeric region of two pairs of small autosomes. The faint hybridization signal on the Y chromosome below the PR could be caused by the heterochromatic nature of the whole Y chromosome [39, 72, 73] pointed out that Bs of some species follow a similar strategy as that proposed for Y chromosome evolution [74], because they are enriched with retrotransposons and other repeated sequences whose accumulation is facilitated by reduced crossing-over, leading to further accumulation of mobile elements.

According to the G banding results (Fig 1A) and DAPI staining (Fig 2A), the subtelomeric regions on four small autosomes that correspond to the B-specific signal are heterochromatic blocks (Fig 1B, Fig 2B). Constitutive heterochromatin was found in the subtelomeric region of four small autosomes in the wood mouse, *Apodemus sylvaticus*, but not in *A*. *flavicollis* [39, 72]. In *A*. *flavicollis* rDNA clusters are located in subtelomeric regions, but such clusters persist in a variable number of chromosomes and usually in up to eight pairs of autosomes [36, 72]. Although rDNA clusters persist on sex chromosomes in *A*. *sylvaticus*, they are not found on sex chromosomes in *A*. *flavicollis* [72].

We were not able to obtain more information about the molecular structure of the subtelomeric regions of the two small pairs of autosomes that were hybridized with the B-specific probe, except that they are heterochromatic and present on Bs. There is a chance that the same type of sequences are located on subtelomeric regions of those autosomes and the PR, and consequently from the PR to Bs. Nevertheless, the presence of such autosomal signals could also indicate a mixed origin of Bs or, more probably, translocation or transposition of repetitive sequences from those autosomal regions to Bs.

Bs could form bivalents among themselves in meiotic divisions in *A*. *flavicollis* [43, 44], although they do not perform any meiotic recombination with the rest of the genome [43]. Restricted recombination with the A set of chromosomes is considered a necessary step for independent evolution of Bs. Despite the unique origin, and/or evolutionary pattern, of all Bs in this species, they probably accumulated changes during evolution which hindered recombination with ancestral chromosomes. At the same time these changes appear to be common for all Bs, as they permit pairing of Bs in meiotic division. Loss of recombination ability with the chromosome of origin, together with different selection pressure on Bs, permits further insertions, the spread and accumulation of mobile elements on other DNA segments [15, 19]. Possible crossing over between Bs shows that, besides origin, additional chromosomes in *A*. *flavicollis* share the same evolutionary pattern. Regardless of the same hybridization affinity and observed pairing in meiotic division, we could still expect slight differences in sequence between Bs from different populations of *A*. *flavicollis*, that are not visible by using FISH, depending on mutation rates, and additionally translocated and amplified segments.

There is evidence that Bs in populations of the same species can differ in origin and DNA content. In *A*. *peninsulae* Bs originate predominantly from autosomes in populations from western Siberia, but in those from the Far East they originate mainly from sex chromosomes [22]. Recent studies of the molecular structure of Bs in *A*. *peninsulae* showed geographic variability in DNA content from distinct populations [23]. We did not detect any differences in hybridization signal between geographically distant samples. All obtained and applied B-specific probes showed the same hybridization pattern strongly indicating a common origin of all B chromosomes in *A*. *flavicollis*. These chromosomes contained the same major repeats with near the same quantity although they could differ in their organization.

Although heterochromatin was considered as an inactive part of the genome for a long time, recent findings showed that it may affect spatial organization in the nucleus and probably successful passage of some meiosis stages [75]. Active heterochromatin was found in Bs of rye [76]. It has been shown that pseudogenes have a function in regulation of the paternal gene, and that they have potential to become genes with new functions [4, 5].

The presence of different gene sequences, whether they are complete or in some stage of degeneration, together with transcriptional activity of several of them, was confirmed in Bs of some species [13, 34, 35]. This evidence implies that the presence of B chromosomes might provide great evolutionary potential for their carriers. In addition, many population studies conducted on *A*. *flavicollis* have confirmed that Bs do have phenotypic effects at different levels [47, 50, 52, 53, 77]. Furthermore, B carriers are considered as better adapted to harsh environmental conditions [50, 78–80]. The observed phenotypic effects, in the absence of a mechanism of accumulation [81], have enabled maintenance of Bs in *A*. *flavicollis* through generations. An origin of Bs from sex chromosomes could be the background of those effects.

## References

[1] Jones RN. B chromosomes in plants. New Phytol. 1995; 131: 411–434. 10.1111/j.1469-8137.1995.tb03079.x 33863119

[2] Camacho JPM, Sharbel TF, Beukeboom LW. B-chromosome evolution. Philos Trans R Soc B Biol Sci. 2000; 355: 163–178. 10.1098/rstb.2000.0556 PMC1692730 10724453

[3] Burt A, Trivers R. Genes in conflict: The biology of selfish genetic elements. Belknap, Cambridge; 2006.

[4] Balakirev ES, Ayala FJ. Pseudogenes: Are they “junk” or functional DNA? Annu Rev Genet. 2003; 37: 123–151. 10.1146/annurev.genet.37.040103.103949 14616058

[5] Pink RC, Wicks K, Caley DP, Punch EK, Jacobs L, Carter DR. Pseudogenes: Pseudo-functional or key regulators in health and disease? RNA. 2011; 17: 792–798. 10.1261/rna.2658311 21398401 PMC3078729

[6] Pennisi E. Genomics encode project writes eulogy for junk DNA. Science. 2012; 337: 1159–1161. 10.1126/science.337.6099.1159 22955811

[7] Shweta M, Vinod G. Repetitive sequences in plant nuclear DNA: types, distribution, evolution and function. Genomics Proteomics Bioinformatics. 2014; 12: 164–171. 10.1016/j.gpb.2014.07.003 25132181 PMC4411372

[8] Palazzo AF, Lee ES. Non-coding RNA: what is functional and what is junk? Frontiers in genetics. 2015; 6: 1–11. 25674102 10.3389/fgene.2015.00002 PMC4306305

[9] Khurana E, Fu Y, Chakravarty D, Demichelis F, Rubin MA, Gerstein M. Role of non-coding sequence variants in cancer. Nature. 2016; 10.1038/nrg.2015.17 26781813

[10] Valente GT, Conte MA, Fantinatti BEA, Cabral-de-Mello DC, Carvalho RF, Vicari MR, et al Origin and evolution of B chromosomes in the cichlid fish *Astatotilapia latifasciata* based on integrated genomic analyses. Mol Biol Evol. 2014; 31: 2061–2072. 10.1093/molbev/msu148 24770715

[11] Jones RN, Rees H. B chromosomes. Academic Press, London, New York; 1982.

[12] Blagojević J, Vujošević M. B chromosomes and developmental homeostasis in the yellow-necked mouse, *Apodemus flavicollis* (Rodentia, Mammalia): Effects on nonmetric traits. Heredity. 2004; 93: 249–254. 10.1038/sj.hdy.6800460 15100709

[13] Makunin AI, Dementyeva PV, Graphodatsky AS, Volobouev VT, Kukekova AV, Trifonov VA. Genes on B chromosomes of vertebrates. Mol Cytogenet. 2014; 7: 1–10. 25538793 10.1186/s13039-014-0099-y PMC4274688

[14] Trifonov VA, Dementyeva PV, Beklemisheva VR, Yudkin DV, Vorobieva NV, Graphodatsky AS. Supernumerary chromosomes, segmental duplications, and evolution. Russ J Genet. 2010; 46: 1094–1096. 21061624

[15] Houben A, Banaei-Moghaddam AM, Klemme S, Timmis JN. Evolution and biology of supernumerary B chromosomes. Cell Mol Life Sci. 2013; 71: 467–478. 10.1007/s00018-013-1437-7 23912901 PMC11113615

[16] Basheva EA, Torgasheva AA, Sakaeva GR, Bidau C, Borodin PM. A- and B-chromosome pairing and recombination in male meiosis of the silver fox (*Vulpes vulpes* L., 1758, Carnivora, Canidae). Chromosome Res. 2010; 18: 689–696. 10.1007/s10577-010-9149-4 20697834

[17] Patton JL. B-chromosome systems in the pocket mouse, *Perognathus baileyi*: meiosis and C-band studies. Chromosoma. 1977; 60: 1–14. 858258 10.1007/BF00330406

[18] Palestis BG, Burt A, Jones RN, Trivers R. B chromosomes are more frequent in mammals with acrocentric karyotypes: support for the theory of centromeric drive. Proc R Soc B Biol Sci. 2004; 271: S22–S24. 10.1098/rsbl.2003.0084 PMC1810000 15101408

[19] Martis MM, Klemme S, Banaei-Moghaddam AM, Blattner FR, Macas J, Schmutzer T, et al Selfish supernumerary chromosome reveals its origin as a mosaic of host genome and organellar sequences. Proc Natl Acad Sci. 2012; 109: 13343–13346. 10.1073/pnas.1204237109 22847450 PMC3421217

[20] Silva DMZ de A, Pansonato-Alves JC, Utsunomia R, Araya-Jaime C, Ruiz-Ruano FJ, Daniel SN, et al Delimiting the origin of a B chromosome by FISH mapping, chromosome painting and DNA sequence analysis in *Astyanax paranae* (Teleostei, Characiformes). PLoS ONE 9:e94896 2014; 10.1371/journal.pone.0094896 24736529 PMC3988084

[21] Ventura K, O’Brien PCM, do Nascimento Moreira C, Yonenaga-Yassuda Y, Ferguson-Smith MA. On the origin and evolution of the extant system of B chromosomes in Oryzomyini radiation (Rodentia, Sigmodontinae). PLOS ONE 10:e0136663 2015; 10.1371/journal.pone.0136663 26305702 PMC4549248

[22] Rubtsov NB, Borisov YuM, Karamysheva TV, Bochkarev MN. The mechanisms of formation and evolution of B chromosomes in Korean field mice *Apodemus peninsulae* (Mammalia, Rodentia), Russ J Genet. 2009; 45: 389–396. 19507698

[23] Rubtsov NB, Kartavtseva IV, Roslik GV, Karamysheva TV, Pavlenko MV, Iwasa MA, et al Features of the B chromosome in Korean field mouse *Apodemus peninsulae* (Thomas, 1906) from Transbaikalia and the Far East identified by the FISH method. Russ J Genet. 2015; 51: 278–288. 26027373

[24] Zhou Q, Zhu HM, Huang QF, Zhao L, Zhang G, Roy SW, et al Deciphering neo-sex and B chromosome evolution by the draft genome of *Drosophila albomicans*. Bmc Genomics. 2012; 13: 109 10.1186/1471-2164-13-109 22439699 PMC3353239

[25] Cheng Y-M, Feng Y-R, Lin Y-P, Peng S-F. Cytomolecular characerization and origin of de novo formed maize B chromosome variants. Chromosome Res. 2016; 24: 183–195. 10.1007/s10577-015-9516-2 26748511

[26] Burgov AG, Karamysheva TV, Rubtsov DN, Andreenkova OV, Rubtsov NB. Comparative FISH analysis of distribution of B chromosomes repetitive DNA in A and B chromosomes in two subspecies of *Podisma sapporensis* (Orthoptera, Acrididae). Cytogenet Genome Res. 2004; 106: 284–288. 10.1159/000079300 15292604

[27] Wurster-Hill DH, Ward OG, Davis BH, Park JP, Moyzis RK, Meyne J,. Fragile sites, telomeric DNA sequences, B chromosomes, and DNA content in raccoon dog, *Nyctereutes procyonoides*,withcomparative notes on foxes, coyote, wolf and raccoon. Cytogenet Genome Res.1988; 49: 278–281. 10.1159/000132677 3150325

[28] Stitou S, Jiménez R, Diaz De La Guardia R, Burgos M. Silent ribosomal cistrons are located at the pairing segment of the postreductional sex chromosomes of *Apodemus sylvaticus* (Rodentia, Muridae). Heredity. 2001; 86: 128–133. 11380657 10.1046/j.1365-2540.2001.00771.x

[29] Poletto AB, Ferreira IA, Martins C. The B chromosomes of the African cichlid fish *Haplochromis obliquidens* harbour 18S rRNA gene copies. BMC Genet. 2010; 11: 1 10.1186/1471-2156-11-1 20051104 PMC2806386

[30] Teruel M, Cabrero J, Perfectti F, Camacho JPM. B chromosomes ancestry revealed byhistone genes in migratory locust. Chromosoma. 2010; 119: 217–225. 10.1007/s00412-009-0251-3 20016909

[31] Graphodatsky AS, Kukekova AV, Yudkin DV, Trifonov VA, Vorobieva NV, Beklemisheva VR, et al The proto-oncogene C-KIT maps to canid B-chromosomes. Chromosome Res. 2005; 13: 113–122. 10.1007/s10577-005-7474-9 15861301

[32] Karamysheva TV, Andreenkova OV, Bochkaerev MN, Borissov YM, Bogdanchikova N, Borodin PM, et al B chromosomes of Korean field mouse *Apodemus peninsulae* (Rodentia, Murinae) analysed by microdissection and FISH. Cytogenet Genome Res. 2002; 96: 154–160. 12438792 10.1159/000063027

[33] Fantinatti BEA, Mazzuchelli J, Valente GT, Cabral-de-Mello DC, Martins C. Genomic content and new insights on the origin of the B chromosome of the cichlid fish *Astatotilapia latifasciata*. Genetica. 2011; 139: 1273–1282. 10.1007/s10709-012-9629-x 22286964

[34] Banaei-Moghaddam AM, Schubert V, Kumke K, Weiβ O, Klemme S, Nagaki K, et al Nondisjunction in favor of a chromosome: The mechanism of rye B chromosome drive during pollen mitosis. Plant Cell. 2012; 24: 4124–4134. 10.1105/tpc.112.105270 23104833 PMC3517240

[35] Trifonov VA, Dementyeva PV, Larkin DM, O’Brien PCM, Perelman PL, Yang F, et al Transcription of a protein-coding gene on B chromosomes of the Siberian roe deer (*Capreolus pygargus*). BMC Biol. 2013; 11: 90 10.1186/1741-7007-11-90 23915065 PMC3751663

[36] Kartavtseva IV. Karyosystematics of wood and field Mice (Rodentia: Muridae). Vladivostok: Dalnauka; 2002.

[37] Ramalhinho MG, Libois R. First report on the presence in France of a B-chromosomes polymorphism in *Apodemus flavicollis*. Mammalia. 2002; 66: 300–303.

[38] Vujošević M, Jojić V, Bugarski-Stanojević V, Blagojević J. Habitat quality and B chromosomes in the yellow-necked mouse *Apodemus flavicollis*. Ital J Zool. 2007; 74: 313–316.

[39] Rovatsos MT, Mitsainas GP, Tryfonopoulos GA, Stamatopoulos C, Giagia-Athanasopoulou EB. A chromosomal study on Greek populations of the genus *Apodemus* (Rodentia, Murinae) reveals new data on B chromosome distribution. Acta Theriol (Warsz). 2008; 53: 157–167.

[40] Wojcik JM, Wojcik AM, Macholan M, Piálek J, Zima J. The mammalian model for population studies of B chromosomes: the wood mouse (*Apodemus*). Cytogenet Genome Res. 2004; 106: 264–270. 10.1159/000079297 15292601

[41] Zima J. Chromosome of certain small mammals from Soutern Bohemia and the Sumava Mts, (CSSR). Folia Zool. 1984; 33: 133–141.

[42] Vujošvić M. B-chromosome polymorphism in *Apodemus flavicollis* (Rodentia, Mammalia) during five years. Caryologia. 1992; 45: 347–352.

[43] Vujošević M, Radosavljević J, Živković S. Meiotic behavior of B chromosomes in yellow necked mouse *Apodemus flavicollis*. Arch Biol Sci. 1990; 42: 39–42.

[44] Banaszek A, Jadwiszczak KA. B-chromosomes behavior during meiosis of yellow-necked mouse, *Apodemus flavicollis*.Folia Zool. 2006; 55: 113–122.

[45] Tanić N, Dedović N, Vujošević M, Dimitrijević B. DNA profiling of B chromosomes from the yellow-necked mouse *Apodemus flavicollis* (Rodentia, Mammalia). Genome Res. 2000; 10: 55–61. 10645950 PMC310505

[46] Tanić N, Vujošević M, Dedović-Tanić N, Dimitrijević B. Differential gene expression in yellow-necked mice *Apodemus flavicollis* (Rodentia, Mammalia) with and without B chromosomes. Chromosoma. 2005; 113: 418–427. 10.1007/s00412-004-0327-z 15657744

[47] Adnađević T, Jovanović VM, Blagojević J, Budinski I, Čabrilo B, Bijelić-Čabriolo O, et al Possible influence of B chromosomes on genes included in immune response and parasite burden in *Apodemus flavicollis*. PLoS ONE 9:e112260 2014; 10.1371/journal.pone.0112260 25372668 PMC4221283

[48] Rajičić M, Adnađević T, Stamenković G, Blagojević J, Vujošević M. Screening of B chromosomes for presence of two genes in yellow-necked mice, *Apodemus flavicollis* (Mammalia, Rodentia). Genetika. 2015; 47: 311–321.

[49] Blagojević J, Vujošević M. Do B chromosomes affect morphometric characters in yellow-necked mice *Apodemus flavicollis* (Rodentia, Mammalia)? Acta Theriol. 2000; 45: 129–135.

[50] Zima J, Piálek J, Macholán M. Possible heterotic effect of B chromosomes on body mass in population of *Apodemus flavicollis*. Can J Zoolog. 2003; 81: 1312–1317.

[51] Blagojević J, Vukićević-Radić O, Vujošević M. B chromosomes and asymmetry of eye lenses in the yellow-necked mouse, *Apodemus flavicollis* (Rodentia, Mammalia). Belg J Zool. 2005; 135: 79–81.

[52] Jojić V, Blagojević J, Ivanović A, Bugarski-Stanojević V, Vujošević M. Morphological integration of the mandible in yellow-necked field mice: the effects of B chromosomes. J Mammal. 2007; 88: 689–695.

[53] Jojić V, Blagojević J, Vujošević M. B chromosomes and cranial variability in yellow-necked field mice (*Apodemus flavicollis*). J Mammal. 2011; 92: 396–406.

[54] Bugarski-Stanojević V, Stamenković G, Blagojević J, Liehr T, Kosyakova N, Rajičić M, et al Exploring supernumeraries- a new marker for screening of B-chromosomes presence in the yellow necked mouse *Apodemus flavicollis* PLoS ONE 11(8): e0161946 2016; 27551940 10.1371/journal.pone.0160946 PMC4994964

[55] Stanyon R, Galleni L. A rapid fibroblast-culture technique for high-resolution karyotypes. B Zool. 1991; 58: 81–3.

[56] Romanenko SA, Biltueva LS, Serdyukova NA, Kulemzina AI, Beklemisheva VR, Gladkikh OL, et al Segmental paleotetraploidy revealed in sterlet (*Acipenser ruthenus*) genome by chromosome painting. Mol Cytogenet. 2015; 10.1186/s13039-015-0194-8 PMC4652396 26587056

[57] Ford CE, Evans EP. Meiotic preparations from mammalian testes In: Benirschke K (ed) Comparative mammalian cytogenetics. Springer, Berlin-Heidelberg-New York; 1966 pp. 461–466.

[58] Hsu TC, Patton JL. Bone marrow preparations for chromosomes studies. Comparative mammalian cytogenetics In: Benirschke K (ed) Comparative mammalian cytogenetics. Springer, Berlin-Heidelberg-New York; 1969 pp. 454–460.

[59] Graphodatsky AS, Radjabli SI. Chromosomes of agricultural and laboratory mammals. Nauka; 1988.

[60] Yang F, Trifonov V, Ng BL, Kosyakova N, Carter NP. Generation of paint probes by flow-sorted and microdissected chromosomes In: Liehr T (ed) Fluorescence *In Situ* Hybridization (FISH)—Application guide. Springer-Verlag Berlin Heidelberg; 2009 pp. 35–52.

[61] Trifonov VA, Vorobieva NN, Rens W. FISH with and without COT1 DNA. In: Liehr T (ed) Fluorescence *In Situ* Hybridization (FISH)—Application Guide. Springer-Verlag Berlin Heidelberg; 2009.

[62] Graphodatsky AS. Comparative chromosomics. Mol Biol. 2007; 41: 408–422. 17685220

[63] M, Blagojević J, Radosavljević J, Bejaković D. B chromosome polymorphism in populations of *Apodemus flavicollis* in Yugoslavia. Genetica. 1991; 83: 167–170.

[64] Sharbel TF, Green DM, Houben A. B chromosome origin in the endemic New Zealand frog *Leiopelma hochstetteri* through sex chromosome devolution. Genome. 1998; 41: 14–22. 9549055 10.1139/gen-41-1-14

[65] Cabrero J, Bakkali M, Bugrov A, Warchalowska-Sliwa E, López-León MD, Perfectti F, et al Multiregional origin of B chromosomes in the grasshopper *Eyprepocnemis plorans*. Chromosoma. 2003; 112: 207–211 10.1007/s00412-003-0264-2 14628147

[66] Rubtsov NB, Karamysheva TV, Bogdanov AS, Likhoshvay TV, Kartavtseva IV. Comparative FISH analysis of C-positive regions of chromosomes of wood mice (Rodentia, Muridae, Sylvaemus). Russ J Genet. 2011; 47: 1096–1110. 22117409

[67] Safronova LD, Cherepanova EV. Behavior of sex chromosomes at early meiosis stages in three wood mice species of the genus *Apodemus* (Rodentia, Muridae). Russ J Genet. 2007; 43: 658–664. 17853806

[68] Stitou S, Guardia de la RD, Jiménez R, Burgos M. Inactive ribosomal cistrons are spread throughout the B chromosomes of *Rattus rattus* (Rodentia, Muridae). Implications for their origin and evolution. Chromosome Res. 2000; 8: 305–311. 10919721 10.1023/a:1009227427575

[69] Flaquer A, Fischer C, Wienker TF. A new sex-specific genetic map of the human pseudoautosomal regions (PAR1 and PAR2). Hum Hered. 2009; 68: 192–200. 10.1159/000224639 19521101

[70] Raudsepp T, Das PJ, Avila F, Chowdhary BP. The pseudoautosomal region and sex chromosome aneuploidies in domestic species. Sex Dev. 2012; 6: 72–83. 10.1159/000330627 21876343

[71] Frédéric B, Yukiko I, Bernard de M. Meiotic recombination in mammals: localization andregulation. Nat Rev Genet. 2013; 14: 794–806. 10.1038/nrg3573 24136506

[72] Gornung E, Cristaldi M, Castiglia R. Comparative cytogenetic analysis of the “Sylvaemus” group of *Apodemus* (Rodentia, Muridae): *A*. *sylvaticus* from Sicily and *A*. *flavicollis* from the central Apennines. Acta Theriol. 2009; 54: 267–275.

[73] Banaei-Moghaddam AM, Martis MM, Macas J, Macas J, Gundlach H, Himmelbach A, et al Genes on B chromosomes: Old questions revisited with new tools. Biochim Biophys Acta BBA—Gene Regul Mech. 2015; 1849: 64–70. 10.1016/j.bbagrm.2014.11.007 25481283

[74] Charlesworth D. Plant sex chromosomes. Genome Dyn. 2008; 4: 83–94. 10.1159/000126008 18756079

[75] Rubtsov NB, Karamysheva TV, Bogdanov AS, Kartavtseva IV, Bochkarev MN, Iwasa MA. Comparative analysis of DNA homology in pericentric regions of chromosomes of wood mice from genera *Apodemus* and *Sylvaemus*. Russ J Genet. 2015; 51: 1233–1242. 27055302

[76] Carchilan M, Delgado M, Ribeiro T, Costa-Nunes P, Caperta A, Morais-Cecílio L, et al Transcriptionally active heterochromatin in rye B chromosomes. Plant Cell. 2007; 19: 1738–1749. 10.1105/tpc.106.046946 17586652 PMC1955731

[77] Blagojević J, Vujošević M. The role of B chromosomes in the population dynamics of yellow-necked wood mice Apodemus flavicollis (Rodentia, Mammalia). Genome. 1995; 38: 472–478. 7557359 10.1139/g95-062

[78] Vujošević M, Blagojević J. Seasonal changes of B-chromosome frequencies within the population of *Apodemus flavicollis* (Rodentia) on Cer mountain in Yugoslavia. Acta Theriol. 1995; 40: 131–137.

[79] Vujošević M, Blagojević J. Does environment affect polymorphism of B chromosomes in the yellow-necked mouse *Apodemus flavicollis*. Z Saugetierkunde. 2000; 65: 313–317.

[80] Blagojević J, Stamenković G, Jojić Šipetić V, Bugarski-Stanojević V, Adnađević T, Vujošević M. B chromosomes in populations of yellow‐necked mice–stowaways or contributing genetic elements? Ital J Zool. 2009; 76: 250–257.

[81] Vujošević M, Živković S. Numerical chromosome polymorphism in *Apodemus flavicollis* and *A*. *sylvaticus* (Mammalia: Rodentia) caused by supernumerary chromosomes. Acta Vet-Beograd. 1987; 37: 81–92.

